# Six months into the war: a first-wave study of stress, anxiety, and depression among in Ukraine

**DOI:** 10.3389/fpsyt.2023.1190465

**Published:** 2023-05-10

**Authors:** Anton Kurapov, Ivan Danyliuk, Andrii Loboda, Argyroula Kalaitzaki, Tobias Kowatsch, Tamara Klimash, Viktoriia Predko

**Affiliations:** ^1^Department of Experimental and Applied Psychology, Faculty of Psychology, Taras Shevchenko National University of Kyiv, Kyiv, Ukraine; ^2^Institute of Medicine, Sumy State University, Sumy, Ukraine; ^3^Department of Social Work, Laboratory of Interdisciplinary Approaches to the Enhancement of Quality of Life, Health Sciences Faculty, Hellenic Mediterranean University, Crete, Greece; ^4^Institute for Implementation Science in Health Care, University of Zürich, Zürich, Switzerland; ^5^School of Medicine, University of St. Gallen, St. Gallen, Switzerland; ^6^Centre for Digital Health Interventions, Department of Management, Technology, and Economics, ETH Zürich, Zürich, Switzerland; ^7^Department of General Psychology, Faculty of Psychology, Taras Shevchenko National university of Kyiv, Kyiv, Ukraine

**Keywords:** war, Ukraine, anxiety, depression, stress, mental health

## Abstract

**Objective:**

This study examines the prevalence and predictors of mental health issues, specifically anxiety, depression, and stress, among Ukrainians during the military conflict with Russia.

**Method:**

A cross-sectional correlational study was conducted six months after the beginning of the conflict. Sociodemographic factors, traumatic experiences, anxiety, depression, and stress were assessed. The study included 706 participants, both men and women, from different age groups and living in various regions of Ukraine. The data were collected from August till October 2022.

**Results:**

The study found that a large portion of the Ukrainian population shows increased levels of anxiety, depression, and stress due to the war. Women were found to be more vulnerable to mental health issues than men, and younger people were found to be more resilient. Worsened financial and employment statuses predicted increased anxiety. Ukrainians who fled the conflict to other countries exhibited higher levels of anxiety, depression, and stress. Direct exposure to trauma predicted increased anxiety and depression, while war-related exposure to “other stressful events” predicted increased acute stress levels.

**Conclusion:**

The findings of this study highlight the importance of addressing the mental health needs of Ukrainians affected by the ongoing conflict. Interventions and support should be tailored to address the specific needs of different groups, particularly women, younger individuals, and those with worsened financial and employment statuses.

## 1. Introduction

War and military conflicts are events that significantly negatively impact all areas of public life, including the mental health of the communities involved. Stress, depression, and anxiety that accompany stressful events associated with war can profoundly affect the physical health of the entire society, its internal relationships, and daily life. Today, it is known that war substantially impacts the occurrence and severity of mental disorders observed in civilians. In particular, the most common conditions include post-traumatic stress disorder (PTSD), depression, and anxiety (1–4). At the same time, the deterioration of pre-existing mental disorders is observed both among people living directly on the territory of the military conflict and among internally displaced persons (IDPs) and refugees (2, 5–9). Most of the existing studies focus on examining the psycho-emotional state and mental health of the affected communities in the post-war periods (1, 2, 4, 10–12). Despite the prevalence of active military conflicts in various parts of the world, our understanding of the effects of such conflicts on the mental health of affected populations, particularly concerning anxiety and stress, remains inadequate. Despite the significance of this issue, few studies have specifically examined the relationship between active military conflict and anxiety and stress levels (13–16).

For this reason, increased research interest is drawn to the situation in Ukraine, where on February 24, 2022, a full-scale military invasion by Russia began. According to the UN, as of November 2022, the number of confirmed deaths among residents of Ukraine amounted to almost 6,500 people, and about 10,000 were injured; however, the actual death toll could be much higher as it is not possible to accurately estimate the number of victims in areas not controlled by the Ukrainian government (17). In addition, more than seven million Ukrainians have become refugees in other countries, and another seven million are registered as IDPs (18). Changes in the usual life course, bereavement, shelling distress, and challenging economic conditions could provoke anxiety disorders among the Ukrainian population. That is why in this research, we answer the following research questions:

1. What is the prevalence and severity of stress, anxiety, and depression among Ukrainians 6 months after the onset of the war?
2. Can sociodemographic factors such as age, gender, and financial and employment status predict levels of stress, anxiety, and depression among Ukrainians?
3. How does proximity to the conflict zone impact levels of stress, anxiety, and depression among Ukrainians?
4. How are stress, anxiety, and depression interrelated among Ukrainians living in regions directly and indirectly affected by military conflict?
5. To what extent do trauma exposure and proximity to the war predict levels of stress, anxiety, and depression among Ukrainians living in regions directly and indirectly affected by military conflict?

## 2. Literature review

Anxiety and stress are expected consequences of traumatic events that affect the quality and safety of a person’s life. War and military conflicts are significant predictors of anxiety disorders since they are characterized by a high degree of trauma, both for mental and physical health (1, 3, 4). The existing research literature shows that war has a significant negative impact on the civilian population involved, causing anxiety (1, 4, 12), stress disorders (6), depression (2, 8), and PTSD (3, 10, 12, 19).

Researchers pay special attention to the issue of the mental health of refugees who are forced to leave their usual residence due to a military conflict in a country or region. In particular, Kashdan et al. (6) confirm that refugees are a vulnerable group and are more prone to psychological distress and anxiety disorders. In turn, Jain et al. (5) have reached similar conclusions, demonstrating an association between refugee status and anxiety disorders, including PTSD and depression. The authors explain the causes of their occurrence as severe changes in the usual way of life, forced relocations, and the need to be separated from relatives and friends. Sangalang et al. (9) show that anxiety disorders in refugees are more common than in immigrants. Refugees adapt to other cultures with more difficulty, and their acculturation is impaired, causing stress and distress. Further, Hameed et al. (11) report that the rate of depression and anxiety among refugees could exceed 40% and sometimes even reach 80%, as was observed among refugees from Cambodia.

The study of the relationship between war and anxiety disorders among the population of Ukraine is still poorly understood. However, few studies provide some insight. Kurapov et al. (13) report that Ukrainian students experience a deterioration in their psycho-emotional state; in particular, a preponderant number of the respondents were depressed and nervous, reporting an increase in their feelings of loneliness and anger; cases of abuse of alcohol, tobacco, or sedatives have also increased among them. Pavlenko et al. (14) focus on the mental health of women employed in education, including female students and faculty members. The study shows that respondents had an increased level of fear and a reduced level of resistance to stressful situations. In this regard, the existing literature emphasizes that there is a link between the war and the anxiety disorders of the civilian population and refugees involved, also in the context of Ukraine. However, there is no understanding of aspects of the mental status of communities and countries that remain in a state of war.

## 3. Materials and methods

### 3.1. Measures

This research was designed as a cross-sectional correlational study with the following independent variables: sociodemographics, proximity to the war zones, and trauma exposure, and dependent variables: anxiety, depression, and stress. The data collection procedure used a demographic questionnaire, i.e., gender, age, marital status, number of children, current place of residence (within or outside Ukraine), current location (never moved within the country, IDP, occupied, and unoccupied territories), employment and financial status, and employment conditions, Patient Health Questionnaire 4 [PHQ-4; (20)] for measuring anxiety and depression (scoring > = 3 indicates the presence of anxiety and depression disorders), Perceived Stress Scale 4 [PSS-4; (21)] for measuring the experience of acute stress, and Life Events Checklist for DSM-5 [LEC-5; (22)] for measuring trauma exposure. The selected scales obtained high reliability scores (Cronbach’s alpha): PHQ-4 [anxiety (*α* = 0.84), depression (*α* = 0.82)], and PSS-4 [stress (*α* = 0.71)].

### 3.2. Participants

The inclusion criteria for the participants were: age 18–65 and being a citizen of Ukraine, either living in different territories of Ukraine or being refugees in other countries. The study did not presume any specific exclusion criteria. The total number of participants who fulfilled the criteria was 706 (age *M* = 32.1), 155 males and 541 females. For estimating the effect size of age, we have outlined the following age groups: youth (*N* = 120, *M* = 18.9, age range 18–20), young adults (*N* = 114, *M* = 22.9, age range 21–25), adults (*N* = 253, *M* = 32.5, age range 26–40), and middle-aged (*N* = 126, *M* = 49.5, age range 41–64).

### 3.3. Data collection

The data for this study were collected from July 20, 2022, until October 2, 2022, *via* an online snowball questionnaire using Google Forms, where participants were asked to share the questionnaire among their family members and friends. Participants were recruited among the students of the faculty of psychology at the Taras Shevchenko National University of Kyiv and at Sumy National University (students were also asked to share the questionnaire among their friends and relatives) through invitations to take part in the study in different Telegram channels, primarily through a news portal called “Hrunt-media” with 60,000+ subscribers. This procedure allowed us to obtain data from participants from various regions of the country, refugees, and IDPs. All questions were presented in the Ukrainian language. Informed consent was obtained from all participants.

### 3.4. Statistical analysis

Statistical analysis was performed using Jamovi (version 2.3.21) and R (version 2022.07.1): (i) To identify the prevalence and severity of stress, anxiety, and depression among Ukrainians, we have used descriptive statistics methods (mean, median, and standard deviation). (ii) To identify correlations among stress, anxiety, and depression, we have used data grouping based on reported current location (never moved within the country, IDP, occupied, and unoccupied territories) using R-package *dplyr*, and Pearson correlation analysis. (iii) To investigate the impact of sociodemographic factors on stress, anxiety, and depression, we performed *t*-test, one- and two-way ANOVA analyses, and estimated the effect size using omega squared (ω^2^). To analyze the association between specific sociodemographic variables, we performed a chi-square test of independence on a contingency table. (iv) To verify the impact of proximity to the conflict zone on stress, anxiety, and depression, we have used one-way ANOVA and estimated the effect size using omega squared (ω^2^). (v) To investigate the extent of the impact of trauma exposure and proximity to the war, we performed one- and two-way ANOVA analyses and estimated the effect size using omega squared (ω^2^).

## 4. Results

### 4.1. Prevalence and severity of anxiety, depression, and stress

Respondents showed average levels of anxiety (*M* = 2.26; Md = 2; SD = 1.7), depression (*M* = 2.45; Md = 2; SD = 1.8), and high levels of perceived stress (*M* = 7.55; Md = 8; SD = 3.16; min = 0; max = 16). Given that the PHQ-4 threshold for anxiety and depression is 3, it was found that 35.5% of the participants showed symptoms of anxiety, and among them, 13.3% had symptoms of severe anxiety. In addition, 42.3% of respondents showed symptoms of depression. Among them, 16.4% of participants showed symptoms of severe depression. In turn, the threshold value on the PSS-4 scale is 6, which means that more than 70% of the surveyed Ukrainians had symptoms of stress, among whom 10.6% of respondents had severe symptoms of stress (from 12 to 16 points). Detailed distribution is presented in Appendix Figure A1.

To compare obtained levels of anxiety, depression, and stress with corresponding measures before the war, we referred to several previous studies (23–26) conducted on healthy population in Ukraine using the same questionnaires (PHQ-4 and PSS-4): detailed comparison is presented in Table 1.

**Table 1:** Comparison of the mean scores of the current and former studies.

Study	Sample size	Description	Measures					
			Anxiety		Depression		Stress	
			M	SD	M	SD	M	SD
(13)	706	Ukrainians (age *M* = 32.1)	2.26	1.7	2.45	1.8	7.55	3.16
(23)	150	Managers (age *M* ≈ 28.3)	2.02	1.61	1.93	1.18		
(24)	532	Visitors of the general medicine clinic (age *M* = 41.9)	–	–	2.14	1.63	–	–
(26)	176	Students (age *M* = 21.1)	–	–	–	–	7.6	2.82
(25)	78	Students (age *M* = 28.5)	–	–	–	–	9.36	1.1

### 4.2. Anxiety, depression, stress, and sociodemographic factors

Independent samples *t*-test was conducted to compare the mean scores of anxiety, depression, and stress between males and females. The results show statistically significant differences in anxiety [*t*(701) = −4.84, *p* < 0.001, Cohen’s *d* = −0.44, 95%] between males (*M* = 1.68, SD = 1.61) and females (*M* = 2.42, SD = 1.69); depression [*t*(701) = −3.95, *p* < 0.001, Cohen’s *d* = −0.36, 95%] between males (*M* = 1.95, SD = 1.69) and females (*M* = 2.59, SD = 1.81); and stress [*t*(701) = −3.74, *p* < 0.001, Cohen’s *d* = −0.34, 95%] between males (*M* = 6.72, SD = 3.2) and females (*M* = 7.78, SD = 3.11).

**Figure 1:**
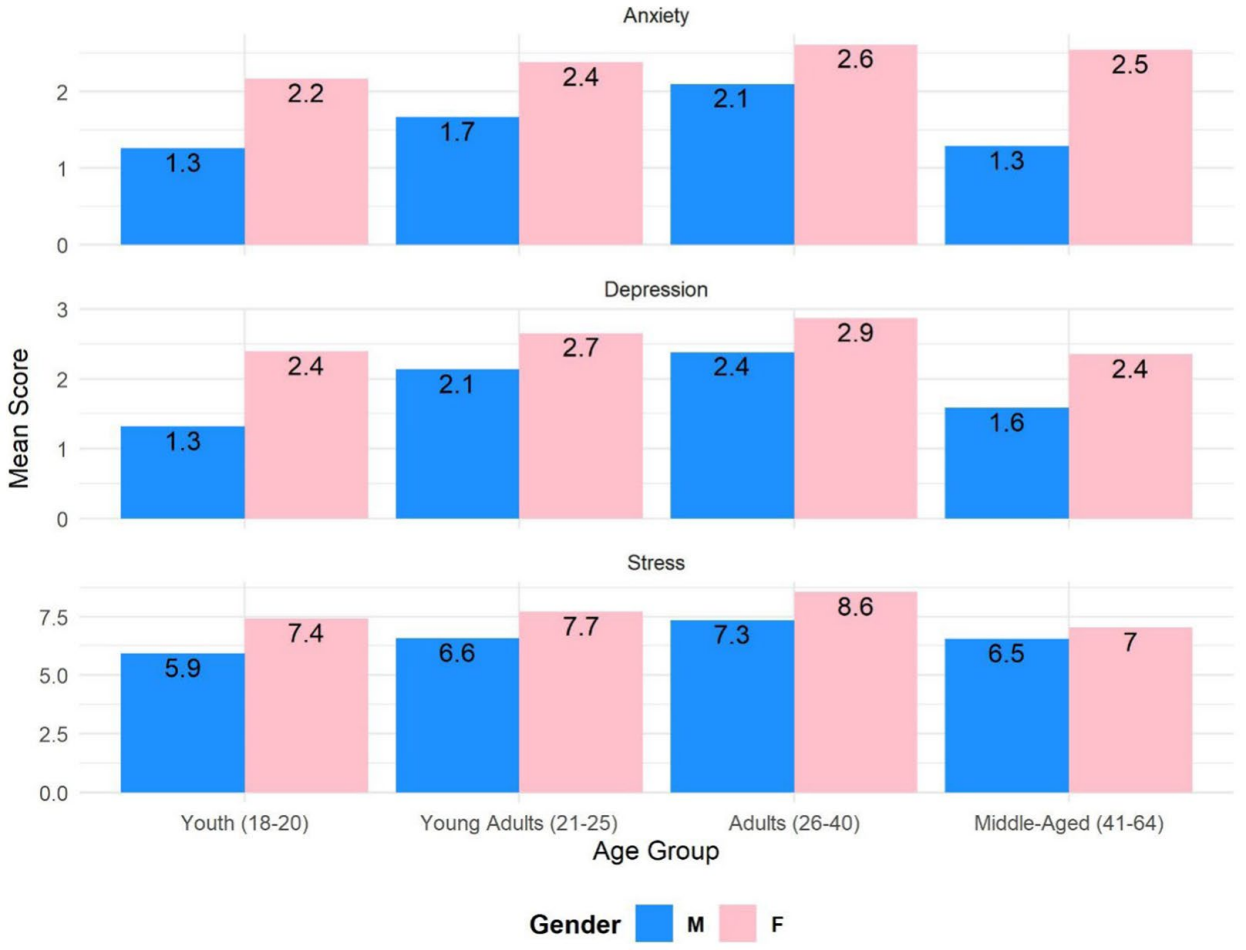
Dependency of anxiety, depression, and stress on age and gender.

A one-way ANOVA was performed to compare the relationships between age groups and anxiety, depression, and stress. There was a statistically significant effect of age groups on anxiety [*F*(3,282) = 4.38, *p* = 0.005, *ω*^2^ = 0.017], in particular, youth (*M* = 1.79, SD = 1.76), young adults (*M* = 2.19, SD = 1.62), adults (*M* = 2.48, SD = 1.68), and middle-aged (*M* = 2.30, SD = 1.68); depression [*F*(3,272) = 3.46, *p* = 0.017, *ω*^2^ = 0.011], in particular, youth (*M* = 2.27, SD = 1.94), young adults (*M* = 2.52, SD = 1.91), adults (*M* = 2.75, SD = 1.65), and middle-aged (*M* = 2.21, SD = 1.85); and stress [*F*(3,277) = 7.48, *p* < 0.001, *ω*^2^ = 0.029], in particular, youth (*M* = 6.97, SD = 3.03), young adults (*M* = 7.40, SD = 3.56), adults (*M* = 8.25, SD = 3.02), and middle-aged (*M* = 6.94, SD = 3.21). Interaction between age and gender did not show statistically significant results (Figure 1).

A one-way ANOVA was used to compare the relationship between financial status and anxiety, depression, and stress. There was a statistically significant effect of financial status on anxiety [*F*(4,123) = 4.06, *p* = 0.004, *ω*^2^ = 0.018], in particular, not enough money even for basic needs [*N* = 50 (7.1%), *M* = 2.92, SD = 1.78], enough money for basic needs only [*N* = 208 (29.6%), *M* = 2.44, SD = 1.72], enough money for basic needs and only critical additional needs [*N* = 288 (41%), *M* = 2.13, SD = 1.71], enough money for basic and some additional needs [*N* = 132 (18.8%), *M* = 1.96, SD = 1.55], enough money for basic and all other additional needs [*N* = 25 (3.6%), *M* = 2.56, SD = 1.69]. The current financial situation is directly linked to employment status [*χ*^2^(24) = 63.6, *p* < 0.001]; specifically, unemployed participants tend to struggle with finances.

A one-way ANOVA was performed to compare the relation among employment statuses and anxiety, depression, and stress. There was a statistically significant effect of employment status on anxiety [*F*(6,59.3) = 3.28, *p* = 0.008, ω^2^ = 0.023], in particular, unemployed (*M* = 2.52, SD = 1.74), governmental employee (*M* = 2.18, SD = 1.47), regular employee (*M* = 2.36, SD = 1.65), freelancer/self-employed/private entrepreneur (*M* = 2.30, SD = 1.69), retired (*M* = 3.42, SD = 1.93), student (*M* = 1.81, SD = 1.71); and depression [*F*(6,60.6) = 2.88, *p* = 0.015, *ω*^2^ = 0.012], in particular, unemployed (*M* = 2.79, SD = 1.87), governmental employee (*M* = 1.85, SD = 1.6), regular employee (*M* = 2.48, SD = 1.74), freelancer/self-employed/private entrepreneur (*M* = 2.48, SD = 1.69), retired (*M* = 1.92, SD = 1.93), student (*M* = 2.24, SD = 1.92).

### 4.3. Distancing from the war

Financial [*χ*^2^(4) = 20.5, *p* < 0.001] and employment [*χ*^2^(6) = 45.9, *p* < 0.001] statuses and directly linked to the current residence within or outside Ukraine: financially secure participants reside in Ukraine, as well as financially insecure, while refugees report similar financial difficulties as Ukrainians, who stayed in Ukraine; overall situation with unemployment is worse within Ukraine.

A one-way ANOVA was used to compare the relationship between current residence within Ukraine and anxiety, depression, and stress. We did not obtain statistically significant results meaning that remaining in any region of Ukraine does not impact anxiety, depression, and stress. Independent samples *t*-test was conducted to compare the mean scores of anxiety, depression, and stress between those who remained in Ukraine and those who left. The results show statistically significant differences in anxiety [*t*(701) = −2.07, *p* = 0.039, Cohen’s *d* = −0.18, 95%] between natives (*M* = 2.18, SD = 1.67) and refugees (*M* = 2.49, SD = 1.78); depression [*t*(701) = −2.22, *p* = 0.027, Cohen’s *d* = −0.19, 95%] between natives (*M* = 2.36, SD = 1.75) and refugees (*M* = 2.71, SD = 1.93); and stress [*t*(701) = −2.97, *p* = 0.003, Cohen’s *d* = −0.26, 95%] between natives (*M* = 7.35, SD = 3.19) and refugees (*M* = 8.16, SD = 2.95).

For the entire sample, a strong positive correlation has been found between anxiety and depression (*r* = 0.612, *p* < 0.001), a strong positive correlation has been found between depression and stress (*r* = 0.566, *p* < 0.001), and a moderate positive correlation has been identified between anxiety and stress (*r* = 0.452, *p* < 0.001). For participants that are closer to the war zones, we obtained a lower correlation coefficient value between anxiety and depression (*r* = 0.596, *p* < 0.001) and between depression and stress (*r* = 0.603, *p* < 0.001), while for those residing further from the war zones correlation between anxiety and depression is higher (*r* = 0.646, *p* < 0.001) and lower between anxiety and stress (*r* = 0.344, *p* < 0.001).

### 4.4. Exposure to traumatic experience and proximity to the war

**Figure 2:**
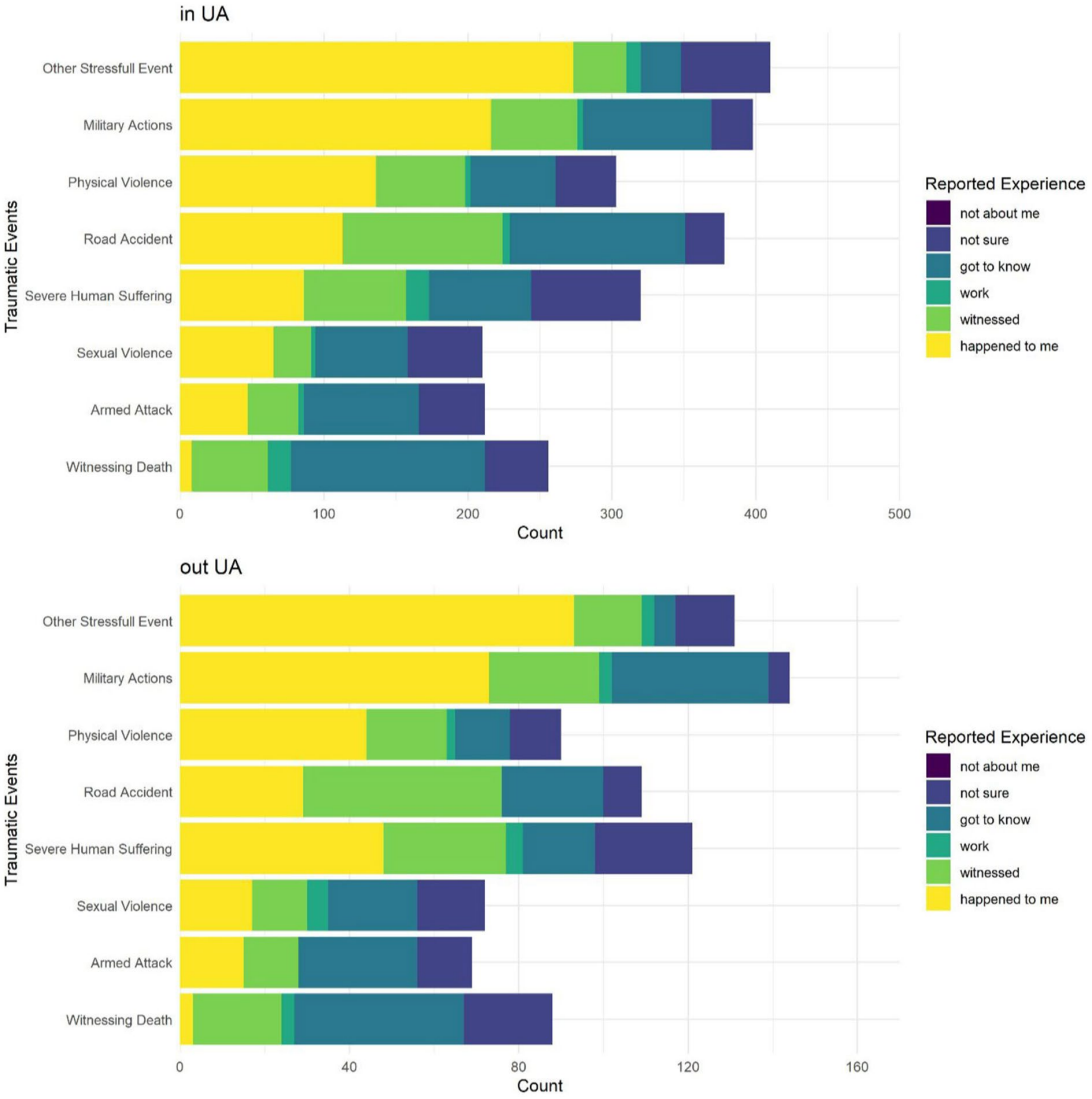
Differentiation of reported trauma exposure for participants living in Ukraine and refugees.

Exposure to traumatic experience was measured with Life Event Checklist for DSM-5. The most prevailing traumatic experiences among Ukrainians include (reported as happened to me) witnessing death (*N* = 11, 1.6%), armed attack (*N* = 62, 8.8%), sexual violence (*N* = 82, 11.6%), severe human suffering (*N* = 134, 19%), road accident (*N* = 143, 20.3%), physical violence (*N* = 181, 25.6%), military actions (*N* = 290, 41.1%), and other stressful events, presumably war-related (*N* = 369, 52.3%). Since we estimated that proximity to the war zone might impact the exposure to trauma, we have divided the sample into two major groups: participants residing in Ukraine and those who have left the country. The chi-square test on relationships between categorical variables did not show statistically significant results [*χ*^2^(5) = 9.96, *p* = 0.076], and the only differences concern road accidents (happened more often in Ukraine) and severe human suffering (happened more often outside of Ukraine). For this reason, it is relevant to assess the differences in mean scores of anxiety, depression, and stress by proximity to the war only for participants who experienced severe human suffering (Figure 2).

A one-way ANOVA was performed to compare the relation between reported trauma exposure experience (armed attack, sexual violence, severe human suffering, physical violence, military actions, and other war-related stressful events) and anxiety, depression, and stress. There was a statistically significant effect of armed attack on anxiety [*F*(5,33.5) = 3.3, *p* = 0.016, *ω*^2^ = 0.014] and depression [*F*(5,30.4) = 3.11, *p* = 0.022, *ω*^2^ = 0.017]; sexual violence on anxiety [*F*(5,61.9) = 5.08, *p* < 0.001, *ω*^2^ = 0.014] and depression [*F*(5,60.7) = 3.19, *p* = 0.013, *ω*^2^ = 0.014]; physical violence on anxiety [*F*(5,50) = 4.33, *p* = 0.002, *ω*^2^ = 0.018] and depression [*F*(5,61.7) = 8.52, *p* < 0.001, *ω*^2^ = 0.024]; military actions on anxiety [*F*(5,56.6) = 2.7, *p* = 0.030, *ω*^2^ = 0.003]; other war-related stressful event anxiety [*F*(5,84.4) = 10.24, *p* < 0.001, *ω*^2^ = 0.059], depression [*F*(5,85.2) = 10.34, *p* < 0.001, *ω*^2^ = 0.062], and stress [*F*(5,83.9) = 5.35, *p* < 0.001, *ω*^2^ = 0.034]. Detailed mean comparisons are presented in Appendix Table A1.

Severe human suffering as reported trauma exposure was assessed for two groups of respondents separately: those residing in Ukraine and outside of Ukraine. The results of one-way ANOVA showed that there was a statistically significant effect of severe human suffering on anxiety [*F*(5,111) = 8.79, *p* < 0.001, *ω*^2^ = 0.072], depression [*F*(5,112) = 6.41, *p* < 0.001, *ω*^2^ = 0.053], and stress [*F*(5,114) = 4.58, *p* < 0.001, *ω*^2^ = 0.033] for participants residing in Ukraine; and on depression [*F*(5,35.4) = 7.44, *p* < 0.001, *ω*^2^ = 0.072] for participants living outside of Ukraine (refugees). Detailed mean comparisons are presented in Appendix Tables A2, A3.

## 5. Discussion and conclusion

### 5.1. Severity of mental health issues

On average, Ukrainians display low levels of anxiety and depression that do not meet the criteria for diagnosis as disorders. However, a significant portion of the population exhibits signs of these disorders. The situation with perceived stress is different, with 70% of Ukrainians reporting acute stress symptoms. Hinz and Schwarz (27) suggest that the prevalence of depression is linked to age and should not exceed 15% for the normal population, indicating that Ukrainians exhibit significantly elevated levels of anxiety and depression due to the war. Although the increased levels of stress among Ukrainians seem self-evident, the results of this study differ from earlier post-war studies (1–6, 9) and are even at odds with recent studies conducted within the first month of the Russian invasion (13–16), which found a significant decline in the mental health of Ukrainians. However, it is important to note that the mental health of individuals who have experienced the stresses of war may deteriorate over time (28). This study does not include socio-cultural variables that could explain the observed outcomes. However, it is possible to assume that the increased national unification in times of crisis could have contributed to the development of efficient coping strategies for overcoming anxiety, depression, and stress among Ukrainians. For example, Ukrainians tend to employ support-seeking and support-giving strategies instead of distancing and emotion-focused strategies, as commonly reported in post-war regions (29).

### 5.2. Sociodemographics and mental health

Our study revealed clear gender and age differences in anxiety, depression, and stress among Ukrainians. Specifically, women showed higher levels of these symptoms than men, consistent with previous research on the association between gender and anxiety disorders (3, 10). Moreover, our results indicate that these symptoms increase with age, except for participants over 40 years old who showed lower levels of anxiety, depression, and stress that are comparable to those of young participants aged 18–20. In contrast, participants between 26 and 40 years old showed the highest levels of anxiety, depression, and stress among all age groups. These findings are consistent with studies on normal populations in non-war zones (27, 30) and suggest that age could predict anxiety, depression, and stress in war zones. Interestingly, our results also indicate that personal control is a significant factor in the relationship between age and mental health. Ross and Mirowsky (31) suggested that personal control increases with age and leads to a decrease in the levels of anxiety, depression, and stress. Although personal control may not be the sole explanation for our findings, older participants may have developed more effective coping mechanisms to deal with stressors, such as the ongoing conflict in Ukraine.

Because of the war, Ukrainians face significant financial challenges, with unemployment and limited income being the main contributors to financial stress. These findings align with previous studies showing a strong link between financial strain and poor mental health outcomes (32). In our study, participants who reported struggling financially or who were unable to cover basic needs showed significantly higher levels of anxiety compared to those who were financially stable. Interestingly, those who reported no financial problems also showed higher anxiety levels. These findings suggest that financial stability may enhance anxiety levels in addition to the war. Additionally, our results indicate that unemployment is a significant predictor of anxiety, with both unemployed and retired participants exhibiting the highest levels of anxiety. These findings align with previous research showing a strong link between unemployment and poor mental health outcomes (33). In terms of depression, we found that it was more common among unemployed participants, as well as governmental and regular employees. Retired participants, on the other hand, reported no cases of depression.

### 5.3. Distancing from the war and trauma experience

We observed that individuals who have remained in their permanent places of residence experience lower levels of anxiety and stress compared to internally displaced individuals and refugees who have left the country. Our findings are in line with previous studies that have reported on the psychological effects of displacement (1–6, 9, 28). The results suggest that Ukrainian individuals consider direct military threats as a less traumatic factor than fleeing their homes and country. The resilience of Ukrainians to military reality and constant threats may be attributed to their family values and attachment to home. However, it is important to note that mental disorders may manifest themselves in the short or long term with a high degree of probability in individuals who have experienced displacement or any other traumatic events.

Participants who reported direct exposure to trauma experience (armed attack, sexual violence, severe human suffering, physical violence, military actions, and other war-related stressful events) show higher levels of anxiety and depression. In particular, it is evident for sexual and physical violence survivals, who report having symptoms of anxiety and depression disorders if this trauma happened to them. Direct experience of a war-related stressful event (reporting that it happened with a participant) significantly increases the signs of symptoms of anxiety and depression disorders, as well as perceived stress. What is more interesting, direct experience of severe human suffering is reported by refugees but not by participants who stayed in Ukraine, which was accompanied by significantly increased levels of anxiety and depression disorders. The situation is completely different for participants who stayed in Ukraine, and they report a slight increase in depression only as a result of experiencing severe human suffering.

The present findings indicate the need for further psychological and psychiatric interventions, as well as future research. One promising avenue for intervention is digital mental health interventions, which have the potential to reach large numbers of individuals in their daily lives (34, 35). These interventions can be scalable and used for screening and identifying those individuals who may require additional human support (36). The results of our study are consistent with earlier research on the mental health of refugees in the Ukrainian context (2, 5, 6, 9). However, we can conclude that the war harmed the mental health of Ukrainians, although it was not as severe as it could have been. It is important to note that the situation may worsen over time, especially after the war.

## 6. Limitations of the study

This study has several limitations. The overall sample may be considered insufficient to represent the entire population; however, considering geographical, age, and other sociodemographic characteristics, this particular sample is capable of showing the main tendencies that are common for entire Ukrainian society 6 months after the beginning of the war, despite women overweighting men. We could not find studies and reports that used PHQ-4 and PSS-4 with a sufficient and representative sample to make a relevant comparison of the mental health situation of Ukrainians before and after the war. The study surveyed general population and did not take into consideration previous history of anxiety, depression, and stress of participants. The study did not examine sociocultural aspects, in particular, the presence of social support within and outside the country, that would allow for an understanding of the reasons for low anxiety and depression since social support might be one of the factors for the majority of the population to maintain low levels of the latter. The study also did not examine the degree of danger in the region where the respondents lived, such as proximity to the front line or the frequency of threats of artillery or missile attacks: we have focused only on the movement within the country and outside of the country. Nevertheless, indicated limitations represent areas for future research topics that would provide an opportunity to deepen the understanding of the impact of war and the socio-cultural context on the clinical picture of the mental health of societies experiencing actual military conflicts.

## Data availability statement

The raw data supporting the conclusions of this article will be made available by the authors, without undue reservation.

## Ethics statement

The studies involving human participants were reviewed and approved by the Ethics Committee of the Hellenic Mediterranean University (87/17-10-2022). The patients/participants provided their written informed consent to participate in this study.

## Author contributions

AKa: conceptualization and methodology of the study. AKu, ID, AL, TKl, and VP: data collection and data proofing. AKu: data analysis. AKu and TKo: manuscript writing. AKu, AKa, and TKo: manuscript review. All authors contributed to the article and approved the submitted version.

## Conflict of interest

TKo is affiliated with the Centre for Digital Health Interventions, a joint initiative of the Institute for Implementation Science in Health Care, University of Zurich, the Department of Management, Technology, and Economics at ETH Zurich, and the Institute of Technology Management and School of Medicine at the University of St. Gallen. Centre for Digital Health Interventions is funded in part by CSS, a Swiss health insurer. TKo was also a cofounder of Pathmate Technologies, a university spin-off company that creates and delivers digital clinical pathways. However, neither CSS nor Pathmate Technologies was involved in this research.

The remaining authors declare that the research was conducted in the absence of any commercial or financial relationships that could be construed as a potential conflict of interest.

## Publisher’s note

All claims expressed in this article are solely those of the authors and do not necessarily represent those of their affiliated organizations, or those of the publisher, the editors and the reviewers. Any product that may be evaluated in this article, or claim that may be made by its manufacturer, is not guaranteed or endorsed by the publisher.

## Supplementary material

The Supplementary material for this article can be found online at: https://www.frontiersin.org/articles/10.3389/fpsyt.2023.1190465/full#supplementary-material
