# Supplementary material for: Six months into the war: a first-wave study of stress, anxiety, and depression among in Ukraine
Source: Front Psychiatry. 2023 May 10;14:1190465. doi: 10.3389/fpsyt.2023.1190465 (PMC10206008; doi:10.3389/fpsyt.2023.1190465)

**Appendix**

Table A1

Mean Score Comparison of Anxiety, Depression, and Stress according to Reported Trauma Exposure

|  | | | | | **Armed Attack** | | | | | | **N** | | | | | | | | | | **Mean** | | | | | | | | | | **SD** | | | | | | | | **SE** | | | | | | | | | | | | | | |  |
| --- | --- | --- | --- | --- | --- | --- | --- | --- | --- | --- | --- | --- | --- | --- | --- | --- | --- | --- | --- | --- | --- | --- | --- | --- | --- | --- | --- | --- | --- | --- | --- | --- | --- | --- | --- | --- | --- | --- | --- | --- | --- | --- | --- | --- | --- | --- | --- | --- | --- | --- | --- | --- | --- | --- |
| Anxiety | | |  | | not about me | | |  | | | 422 | | | | |  | | | | | 2.07 | | | | |  | | | | | 1.673 | | | |  | | | | 0.0815 | | | | |  | | | | | | | | | |  |
|  | | |  | | not sure | | |  | | | 59 | | | | |  | | | | | 2.59 | | | | |  | | | | | 1.609 | | | |  | | | | 0.2095 | | | | |  | | | | | | | | | |  |
|  | | |  | | got to know | | |  | | | 108 | | | | |  | | | | | 2.49 | | | | |  | | | | | 1.683 | | | |  | | | | 0.1619 | | | | |  | | | | | | | | | |  |
|  | | |  | | work | | |  | | | 4 | | | | |  | | | | | 1.75 | | | | |  | | | | | 0.500 | | | |  | | | | 0.2500 | | | | |  | | | | | | | | | |  |
|  | | |  | | witnessed | | |  | | | 48 | | | | |  | | | | | 2.69 | | | | |  | | | | | 1.858 | | | |  | | | | 0.2682 | | | | |  | | | | | | | | | |  |
|  | | |  | | happened to me | | |  | | | 62 | | | | |  | | | | | 2.53 | | | | |  | | | | | 1.808 | | | |  | | | | 0.2296 | | | | |  | | | | | | | | | |  |
| Depression | | |  | | not about me | | |  | | | 422 | | | | |  | | | | | 2.23 | | | | |  | | | | | 1.790 | | | |  | | | | 0.0871 | | | | |  | | | | | | | | | |  |
|  | | |  | | not sure | | |  | | | 59 | | | | |  | | | | | 2.71 | | | | |  | | | | | 1.587 | | | |  | | | | 0.2066 | | | | |  | | | | | | | | | |  |
|  | | |  | | got to know | | |  | | | 108 | | | | |  | | | | | 2.81 | | | | |  | | | | | 1.675 | | | |  | | | | 0.1612 | | | | |  | | | | | | | | | |  |
|  | | |  | | work | | |  | | | 4 | | | | |  | | | | | 1.75 | | | | |  | | | | | 2.062 | | | |  | | | | 1.0308 | | | | |  | | | | | | | | | |  |
|  | | |  | | witnessed | | |  | | | 48 | | | | |  | | | | | 2.79 | | | | |  | | | | | 1.978 | | | |  | | | | 0.2855 | | | | |  | | | | | | | | | |  |
|  | | |  | | happened to me | | |  | | | 62 | | | | |  | | | | | 2.81 | | | | |  | | | | | 1.949 | | | |  | | | | 0.2475 | | | | |  | | | | | | | | | |  |
|  | | | | | | | | | | | | | | | | | | | | | | | | | | | | | | | | | | | | | | | | | | | | | | | | | | | | | |  |
|  | | | | | **Sexual Violence** | | | | | | | | | **N** | | | | | | | | **Mean** | | | | | | | | | | **SD** | | | | | | | | | | | **SE** | | | | | | | | | | | |
| Anxiety | | |  | | not about me | | | |  | | | | | 421 | | | | |  | | | 2.04 | | | | |  | | | | | 1.641 | | | |  | | | | | | 0.0800 | | | | | |  | | | | |  |  |
|  | | |  | | not sure | | | |  | | | | | 68 | | | | |  | | | 2.53 | | | | |  | | | | | 1.816 | | | |  | | | | | | 0.2202 | | | | | |  | | | | |  |  |
|  | | |  | | got to know | | | |  | | | | | 85 | | | | |  | | | 2.60 | | | | |  | | | | | 1.767 | | | |  | | | | | | 0.1917 | | | | | |  | | | | |  |  |
|  | | |  | | work | | | |  | | | | | 8 | | | | |  | | | 1.38 | | | | |  | | | | | 0.916 | | | |  | | | | | | 0.3239 | | | | | |  | | | | |  |  |
|  | | |  | | witnessed | | | |  | | | | | 39 | | | | |  | | | 2.59 | | | | |  | | | | | 1.817 | | | |  | | | | | | 0.2909 | | | | | |  | | | | |  |  |
|  | | |  | | happened to me | | | |  | | | | | 82 | | | | |  | | | 2.72 | | | | |  | | | | | 1.680 | | | |  | | | | | | 0.1855 | | | | | |  | | | | |  |  |
| Depression | | |  | | not about me | | | |  | | | | | 421 | | | | |  | | | 2.26 | | | | |  | | | | | 1.810 | | | |  | | | | | | 0.0882 | | | | | |  | | | | |  |  |
|  | | |  | | not sure | | | |  | | | | | 68 | | | | |  | | | 2.68 | | | | |  | | | | | 1.714 | | | |  | | | | | | 0.2079 | | | | | |  | | | | |  |  |
|  | | |  | | got to know | | | |  | | | | | 85 | | | | |  | | | 2.91 | | | | |  | | | | | 1.652 | | | |  | | | | | | 0.1792 | | | | | |  | | | | |  |  |
|  | | |  | | work | | | |  | | | | | 8 | | | | |  | | | 1.88 | | | | |  | | | | | 1.356 | | | |  | | | | | | 0.4795 | | | | | |  | | | | |  |  |
|  | | |  | | witnessed | | | |  | | | | | 39 | | | | |  | | | 2.54 | | | | |  | | | | | 1.931 | | | |  | | | | | | 0.3092 | | | | | |  | | | | |  |  |
|  | | |  | | happened to me | | | |  | | | | | 82 | | | | |  | | | 2.78 | | | | |  | | | | | 1.833 | | | |  | | | | | | 0.2024 | | | | | |  | | | | |  |  |
|  | | | | | | | | | | | | | | | | | | | | | | | | | | | | | | | | | | | | | | | | | | | | | | | | | | | | | | |
|  | | | | | **Physical Violence** | | | | | | | **N** | | | | | | | | | | | **Mean** | | | | | | | | | **SD** | | | | | | | | | **SE** | | | | | | | | | | |  |  |  |
| Anxiety | | |  | | not about me | | | | |  | | 310 | | | | |  | | | | | | 2.065 | | | | |  | | | | 1.677 | | | |  | | | | | 0.0952 | | | | | | |  | | | |  |  |  |
|  | | |  | | not sure | | | | |  | | 54 | | | | |  | | | | | | 2.815 | | | | |  | | | | 1.614 | | | |  | | | | | 0.2197 | | | | | | |  | | | |  |  |  |
|  | | |  | | got to know | | | | |  | | 72 | | | | |  | | | | | | 2.653 | | | | |  | | | | 1.809 | | | |  | | | | | 0.2132 | | | | | | |  | | | |  |  |  |
|  | | |  | | work | | | | |  | | 6 | | | | |  | | | | | | 1.000 | | | | |  | | | | 1.095 | | | |  | | | | | 0.4472 | | | | | | |  | | | |  |  |  |
|  | | |  | | witnessed | | | | |  | | 81 | | | | |  | | | | | | 2.148 | | | | |  | | | | 1.509 | | | |  | | | | | 0.1677 | | | | | | |  | | | |  |  |  |
|  | | |  | | happened to me | | | | |  | | 180 | | | | |  | | | | | | 2.361 | | | | |  | | | | 1.765 | | | |  | | | | | 0.1315 | | | | | | |  | | | |  |  |  |
| Depression | | |  | | not about me | | | | |  | | 310 | | | | |  | | | | | | 2.174 | | | | |  | | | | 1.778 | | | |  | | | | | 0.1010 | | | | | | |  | | | |  |  |  |
|  | | |  | | not sure | | | | |  | | 54 | | | | |  | | | | | | 2.685 | | | | |  | | | | 1.576 | | | |  | | | | | 0.2145 | | | | | | |  | | | |  |  |  |
|  | | |  | | got to know | | | | |  | | 72 | | | | |  | | | | | | 2.917 | | | | |  | | | | 1.701 | | | |  | | | | | 0.2005 | | | | | | |  | | | |  |  |  |
|  | | |  | | work | | | | |  | | 6 | | | | |  | | | | | | 0.667 | | | | |  | | | | 0.816 | | | |  | | | | | 0.3333 | | | | | | |  | | | |  |  |  |
|  | | |  | | witnessed | | | | |  | | 81 | | | | |  | | | | | | 2.679 | | | | |  | | | | 1.809 | | | |  | | | | | 0.2009 | | | | | | |  | | | |  |  |  |
|  | | |  | | happened to me | | | | |  | | 180 | | | | |  | | | | | | 2.617 | | | | |  | | | | 1.874 | | | |  | | | | | 0.1397 | | | | | | |  | | | |  |  |  |
|  | | | | | | | | | | | | | | | | | | | | | | | | | | | | | | | | | | | | | | | | | | | | | | | | | | | | | | |
|  | | **Military Actions** | | | | | | | | | | | **N** | | | | | | | | | | | **Mean** | | | | | | | | | **SD** | | | | | | | **SE** | | | | | | | | | | |  |  |  |  |
| Anxiety |  | not about me | | | |  | | | | | | | 161 | | | | |  | | | | | | 2.124 | | | | | |  | | | 1.78 | | | |  | | | 0.1404 | | | | | |  | | | | |  |  |  |  |
|  |  | not sure | | | |  | | | | | | | 34 | | | | |  | | | | | | 2.471 | | | | | |  | | | 2.08 | | | |  | | | 0.3563 | | | | | |  | | | | |  |  |  |  |
|  |  | got to know | | | |  | | | | | | | 126 | | | | |  | | | | | | 2.246 | | | | | |  | | | 1.58 | | | |  | | | 0.1411 | | | | | |  | | | | |  |  |  |  |
|  |  | work | | | |  | | | | | | | 7 | | | | |  | | | | | | 0.857 | | | | | |  | | | 1.07 | | | |  | | | 0.4041 | | | | | |  | | | | |  |  |  |  |
|  |  | witnessed | | | |  | | | | | | | 86 | | | | |  | | | | | | 2.244 | | | | | |  | | | 1.69 | | | |  | | | 0.1819 | | | | | |  | | | | |  |  |  |  |
|  |  | happened to me | | | |  | | | | | | | 289 | | | | |  | | | | | | 2.353 | | | | | |  | | | 1.67 | | | |  | | | 0.0983 | | | | | |  | | | | |  |  |  |  |
|  | | | | | | | | | | | | | | | | | | | | | | | | | | | | | | | | | | | | | | | | | | | | | | | | |  |  |  |  |  |  |
|  | | | | **Any other Stressful Event** | | | | | | | | | | | **N** | | | | | | | | | | **Mean** | | | | | | | | | **SD** | | | | | | | | **SE** | | | | | | | |  |  |  |  |  |
| Anxiety | | |  | not about me | | |  | | | | | | | | 162 | | | | |  | | | | | 1.73 | | | |  | | | | | 1.55 | | | |  | | | | 0.1220 | | |  | | | | |  |  |  |  |  |
|  | | |  | not sure | | |  | | | | | | | | 76 | | | | |  | | | | | 1.64 | | | |  | | | | | 1.31 | | | |  | | | | 0.1507 | | |  | | | | |  |  |  |  |  |
|  | | |  | got to know | | |  | | | | | | | | 33 | | | | |  | | | | | 2.58 | | | |  | | | | | 1.56 | | | |  | | | | 0.2719 | | |  | | | | |  |  |  |  |  |
|  | | |  | work | | |  | | | | | | | | 13 | | | | |  | | | | | 1.77 | | | |  | | | | | 1.74 | | | |  | | | | 0.4824 | | |  | | | | |  |  |  |  |  |
|  | | |  | witnessed | | |  | | | | | | | | 53 | | | | |  | | | | | 2.09 | | | |  | | | | | 1.38 | | | |  | | | | 0.1891 | | |  | | | | |  |  |  |  |  |
|  | | |  | happened to me | | |  | | | | | | | | 366 | | | | |  | | | | | 2.63 | | | |  | | | | | 1.79 | | | |  | | | | 0.0937 | | |  | | | | |  |  |  |  |  |
| Depression | | |  | not about me | | |  | | | | | | | | 162 | | | | |  | | | | | 1.89 | | | |  | | | | | 1.83 | | | |  | | | | 0.1436 | | |  | | | | |  |  |  |  |  |
|  | | |  | not sure | | |  | | | | | | | | 76 | | | | |  | | | | | 1.92 | | | |  | | | | | 1.48 | | | |  | | | | 0.1694 | | |  | | | | |  |  |  |  |  |
|  | | |  | got to know | | |  | | | | | | | | 33 | | | | |  | | | | | 2.85 | | | |  | | | | | 1.52 | | | |  | | | | 0.2652 | | |  | | | | |  |  |  |  |  |
|  | | |  | work | | |  | | | | | | | | 13 | | | | |  | | | | | 1.85 | | | |  | | | | | 1.46 | | | |  | | | | 0.4058 | | |  | | | | |  |  |  |  |  |
|  | | |  | witnessed | | |  | | | | | | | | 53 | | | | |  | | | | | 1.96 | | | |  | | | | | 1.54 | | | |  | | | | 0.2121 | | |  | | | | |  |  |  |  |  |
|  | | |  | happened to me | | |  | | | | | | | | 366 | | | | |  | | | | | 2.86 | | | |  | | | | | 1.81 | | | |  | | | | 0.0946 | | |  | | | | |  |  |  |  |  |
| Stress | | |  | not about me | | |  | | | | | | | | 162 | | | | |  | | | | | 6.54 | | | |  | | | | | 3.43 | | | |  | | | | 0.2692 | | |  | | | | |  |  |  |  |  |
|  | | |  | not sure | | |  | | | | | | | | 76 | | | | |  | | | | | 7.47 | | | |  | | | | | 2.95 | | | |  | | | | 0.3385 | | |  | | | | |  |  |  |  |  |
|  | | |  | got to know | | |  | | | | | | | | 33 | | | | |  | | | | | 7.24 | | | |  | | | | | 2.36 | | | |  | | | | 0.4106 | | |  | | | | |  |  |  |  |  |
|  | | |  | work | | |  | | | | | | | | 13 | | | | |  | | | | | 7.23 | | | |  | | | | | 3.49 | | | |  | | | | 0.9684 | | |  | | | | |  |  |  |  |  |
|  | | |  | witnessed | | |  | | | | | | | | 53 | | | | |  | | | | | 7.15 | | | |  | | | | | 2.99 | | | |  | | | | 0.4107 | | |  | | | | |  |  |  |  |  |
|  | | |  | happened to me | | |  | | | | | | | | 366 | | | | |  | | | | | 8.10 | | | |  | | | | | 3.04 | | | |  | | | | 0.1588 | | |  | | | | |  |  |  |  |  |
|  | | | | | | | | | | | | | | | | | | | | | | | | | | | | | | | | | | | | | | | | | | | | | | |  |  |  |  |  |  |  |  |

Table A2

Mean Score Comparison of Anxiety, Depression, and Stress according to Severe Human Suffering for Participants Residing in Ukraine

|  | | **Severe Human Suffering** | | **N** | | **Mean** | | **SD** | | **SE** | |
| --- | --- | --- | --- | --- | --- | --- | --- | --- | --- | --- | --- |
| Anxiety |  | not about me |  | 210 |  | 1.83 |  | 1.61 |  | 0.111 |  |
|  |  | not sure |  | 76 |  | 1.78 |  | 1.44 |  | 0.165 |  |
|  |  | got to know |  | 71 |  | 2.38 |  | 1.62 |  | 0.192 |  |
|  |  | work |  | 16 |  | 2.44 |  | 2.00 |  | 0.500 |  |
|  |  | witnessed |  | 71 |  | 2.25 |  | 1.63 |  | 0.193 |  |
|  |  | happened to me |  | 86 |  | 3.13 |  | 1.66 |  | 0.180 |  |
| Depression |  | not about me |  | 210 |  | 1.99 |  | 1.78 |  | 0.123 |  |
|  |  | not sure |  | 76 |  | 2.17 |  | 1.55 |  | 0.178 |  |
|  |  | got to know |  | 71 |  | 2.66 |  | 1.37 |  | 0.163 |  |
|  |  | work |  | 16 |  | 2.56 |  | 1.93 |  | 0.483 |  |
|  |  | witnessed |  | 71 |  | 2.30 |  | 1.75 |  | 0.208 |  |
|  |  | happened to me |  | 86 |  | 3.21 |  | 1.81 |  | 0.195 |  |
| Stress |  | not about me |  | 210 |  | 6.66 |  | 3.52 |  | 0.243 |  |
|  |  | not sure |  | 76 |  | 7.37 |  | 2.75 |  | 0.315 |  |
|  |  | got to know |  | 71 |  | 7.56 |  | 2.81 |  | 0.333 |  |
|  |  | work |  | 16 |  | 7.56 |  | 2.94 |  | 0.736 |  |
|  |  | witnessed |  | 71 |  | 7.68 |  | 3.07 |  | 0.364 |  |
|  |  | happened to me |  | 86 |  | 8.51 |  | 2.82 |  | 0.304 |  |
|  | | | | | | | | | | | |

 Table A3

Mean Score Comparison of Depression according to Severe Human Suffering for Participants Residing outside of Ukraine (refugees)

|  | | **Severe Human Suffering** | | **N** | | **Mean** | | **SD** | | **SE** | |
| --- | --- | --- | --- | --- | --- | --- | --- | --- | --- | --- | --- |
| Depression |  | not about me |  | 52 |  | 2.12 |  | 1.854 |  | 0.257 |  |
|  |  | not sure |  | 23 |  | 2.26 |  | 1.711 |  | 0.357 |  |
|  |  | got to know |  | 17 |  | 2.76 |  | 1.602 |  | 0.389 |  |
|  |  | work |  | 4 |  | 1.25 |  | 0.500 |  | 0.250 |  |
|  |  | witnessed |  | 29 |  | 3.03 |  | 1.861 |  | 0.346 |  |
|  |  | happened to me |  | 48 |  | 3.48 |  | 2.042 |  | 0.295 |  |
|  | | | | | | | | | | | |

**Figure A1**

*Distribution of Obtained Anxiety, Depression, and Stress Scores*


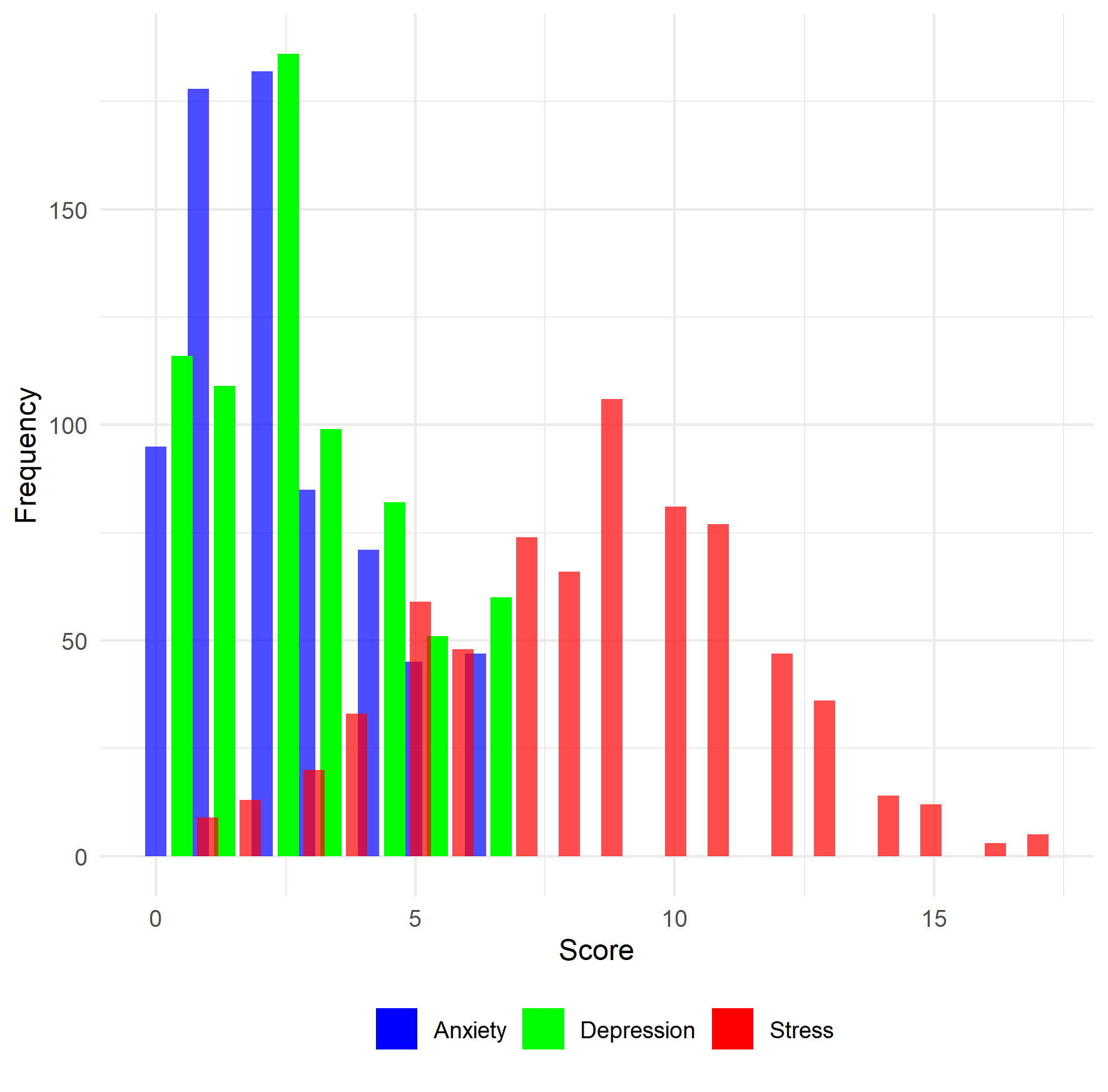

Supplement: Supplementary file 1 [file Data_Sheet_1.docx]
